# Evaluating intervention strategies in controlling coronavirus disease 2019 (COVID-19) spread in care homes: An agent-based model

**DOI:** 10.1017/ice.2020.1369

**Published:** 2020-12-14

**Authors:** Le L. K. Nguyen, Susan Howick, Dennis McLafferty, Gillian H. Anderson, Sahaya J. Pravinkumar, Robert Van Der Meer, Itamar Megiddo

**Affiliations:** 1 Department of Management Science, Strathclyde Business School, University of Strathclyde, Glasgow, United Kingdom; 2 Adult Services, Health & Social Care North Lanarkshire, Motherwell, United Kingdom; 3 Department of Public Health, NHS Lanarkshire, Kirklands Hospital, Bothwell, United Kingdom

## Abstract

**Background:**

Care homes are vulnerable to widespread transmission of severe acute respiratory coronavirus virus 2 (SARS-CoV-2) with poor outcomes for staff and residents. Infection control interventions in care homes need to not only be effective in containing the spread of coronavirus disease 2019 (COVID-19) but also feasible to implement in this special setting which is both a healthcare institution and a home.

**Methods:**

We developed an agent-based model that simulates the transmission dynamics of COVID-19 via contacts between individuals, including residents, staff members, and visitors in a care home setting. We explored a representative care home in Scotland in our base case and explore other care home setups in an uncertainty analysis. We evaluated the effectiveness of a range of intervention strategies in controlling the spread of COVID-19.

**Results:**

In the presence of the reference interventions that have been implemented in many care homes, including testing of new admissions, isolation of symptomatic residents, and restricted public visiting, routine testing of staff appears to be the most effective and practical approach. Routine testing of residents is no more effective as a reference strategy while routine testing of both staff and residents only shows a negligible additive effect. Modeling results are very sensitive to transmission probability per contact, but the qualitative finding is robust to varying parameter values in our uncertainty analysis.

**Conclusions:**

Our model predictions suggest that routine testing should target staff in care homes in conjunction with adherence to strict hand hygiene and using personal protective equipment to reduce risk of transmission per contact.

As of July 15, 2020, ~24 million people had been infected with severe acute respiratory coronavirus virus 2 (SARS-CoV-2) worldwide, and 3.4% of those infected have died.^1^ Many studies have demonstrated that comorbidity and old age are associated with poor outcomes among coronavirus disease 2019 (COVID-19) patients.^2,3^ Care homes across the globe, where most residents are elderly and have complex medical and care needs, have suffered devastating outcomes.^4,5^ High rates of mortality involving COVID-19 have been attributed to residents in this setting (eg, 40%–70% in Canada,^6^ 33%–64% in European countries,^5^ and 47% in Scotland^7^).

Care homes are integral to the wider healthcare system, and it is essential that they continue to function safely and effectively amid the COVID-19 pandemic to avoid increasing the pressure on the acute-care sector. If care homes stop admitting patients discharged from hospitals, patients have to stay in hospitals longer than they need, putting them at greater risk, adding to the pressure on hospitals, and causing tremendous distress for many individuals. Because vaccination for COVID-19 is currently unavailable, infection control interventions within care homes and across settings are vitally important to protect the vulnerable residents and healthcare workers.

Agent-based models (ABMs) have been used to study epidemic behavior and interventions and to mitigate them in the past couple of decades, leading to new insights. Although compartmental models have been popularly used to simulate transmission dynamics of infectious diseases at the population level, ABMs have been useful for understanding the effects of heterogeneous individual characteristics and behavior in conjunction with the stochasticity of transmission events. They have shown that individual characteristics such as age, comorbidity, and socio-spatial structures and contact patterns in different settings influence disease spread and the effectiveness of interventions.^8–12^ Many of these factors are important in the care-home setting and for COVID-19 specifically. Influenza and COVID-19 pandemic ABMs have studied disease dynamics and interventions at the population level,^10,13–15^ and a number of recent ABMs have investigated how interventions in small-scale settings, such as schools and workplaces, would influence the behavior of epidemics in wider communities.^10,16,17^ None of these models explores the care-home setting, and many measures implemented schools and workplaces, such as closure and social distancing, are not suitable for care homes that also act as a residence and where staff interaction with residents is often unavoidable.

Many studies have investigated the spread of SARS-CoV-2 in the general population, but research on the unique transmission dynamics and interventions for COVID-19 in healthcare settings, and care homes in particular, is scarce. We searched PubMed, MedRxiv, and BioRxiv for papers published between January 1, 2020, and July 15, 2020, that contained the terms (COVID OR coronavirus OR nCoV OR SARS-CoV-2) AND (care home* OR nursing home* OR skilled nursing facility* OR long-term care OR LTCF* OR residential care). We identified 152 preprints and articles published in academic journals, mostly outbreak reports, point prevalence surveys, commentary and editorial papers that discuss the importance and challenges of controlling the spread of SARS-CoV-2 in this setting. They describe experiences of containing spread in some specific care homes and a need for improved control interventions, and they call for more attention and a plan from governments. We found 1 preprint modeling study^18^ that evaluates the capability of surveillance strategies to detect simulated outbreaks under limited testing capacity in a long-term care hospital. This paucity suggests a lack of research on the transmission dynamics of SARS-CoV-2 and the effectiveness of infection control interventions in this setting. Therefore, we investigated the transmission dynamics of SARS-CoV-2 in a care home and the effectiveness of a range of infection control intervention strategies using agent-based modeling.

## Methods

### Model

**Fig. 1.**
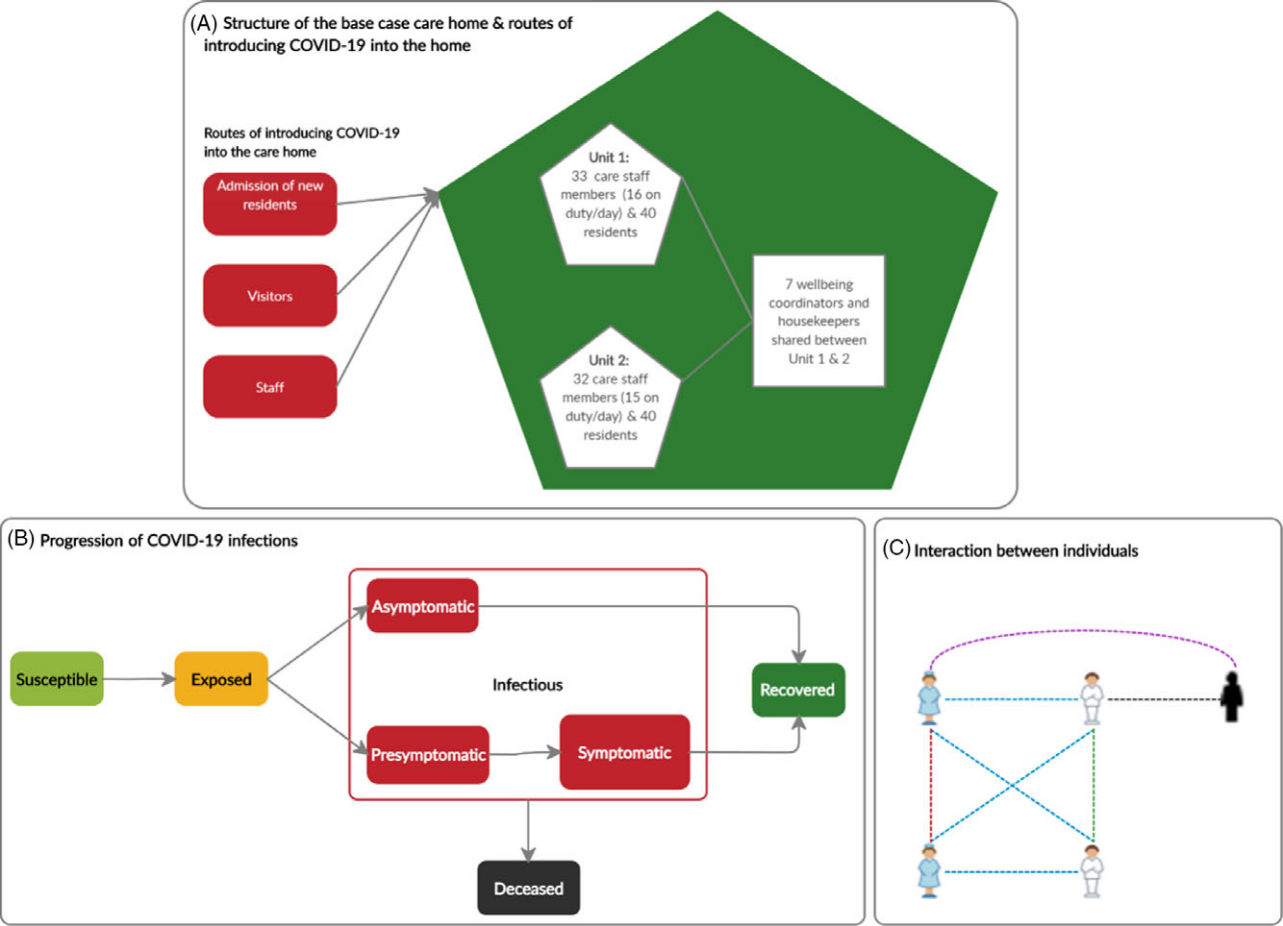
Overview of the model structure. (A) The structure of the care home and routes of introducing SARS-CoV-2 into the home. The base case care home, representative of a care home in North Lanarkshire, Scotland, has 80 residents and a team of 72 staff members. It is split into 2 units containing 40 residents and 16 and 15 care staff members on duty per unit per day. The staff pools for the 2 units contain 33 and 32 care staff members respectively. A group of 7 well-being coordinators and housekeepers is shared between the 2 units. (B) The progression of COVID-19 cases. Susceptible people may acquire the infection when exposed to infectious sources. They are infected but not yet infectious (exposed state). Once exposed people become infectious, they can either remain asymptomatic for the entire infectious period or develop symptoms after a pre-symptomatic period. Symptoms could be mild or severe and require hospitalizations. Infectious people will eventually recover or die. (C) Interactions between residents, staff and visitors in a care home. The dashed lines linking individuals denote their possible ways of interaction. Different colours are used for these lines to distinguish different types of interaction: blue, staff-resident interaction; green, resident-resident interaction; red, staff–staff interaction; black, resident-visitor interaction; and purple, staff–visitor interactions.

We developed an ABM that simulates the transmission dynamics of SARS-CoV-2 via contacts between individual agents, including residents, staff members, and visitors within a care home. We assumed that all rooms are single occupancy because the vast majority of rooms in Scottish care homes are of this type^19^ and, based on discussions with care-home stakeholders, single occupancy has been a consensus practice during the COVID-19 pandemic. The model structure (Fig. 1) is described in detail in the ‘ODD’ (overview, design concepts, and details) protocol (Appendix S1 online). In brief, susceptible individuals may acquire the infection when exposed to infectious sources. They are infected but not yet infectious (exposed). Once exposed, individuals become infectious, they can either remain asymptomatic for the entire infectious period or develop symptoms after a presymptomatic period. Symptoms could be mild or severe and require hospitalization. Infectious individuals eventually recover or die. Infections can be imported into the care home by infected residents upon admission (from hospitals and the community) and staff or visitors who acquired the infection elsewhere. The COVID-19 prevalence in hospitals and the community determine the probability at which these individuals introduce the infection into the care home. Transmission events occur through contacts made between susceptible and infectious (presymptomatic, asymptomatic, and symptomatic) individuals at risk, determined by the infection probability per contact. We assumed that recovered individuals are immune to reinfection throughout the simulated time. As we examined the spread of SARS-CoV-2 once infection already exists in the care home, 1 random resident is infected at the beginning of the simulation; staff and other residents are susceptible.

### Data collection and parameters

We interviewed care-home stakeholders including managers, staff in different roles, and we had regular discussions with representatives from the Health and Social Care Partnerships and Public Health in Lanarkshire to analyze the problem, build the model, and design the intervention strategies. The interviews were semistructured, and each lasted ~45–60 minutes. We also conducted literature reviews to obtain the values for parameters characterizing the transmission of SARS-CoV-2 and the disease progression. Other parameters are based on national data for Scotland and regional data for North Lanarkshire where available. Model input parameters used for the base case simulation are presented in Table 1.

**Table 1.** Input Parameters for Base Case Simulation and Distributions of Parameters

Input Parameter	Base Case Value	Sensitivity/Uncertainty Analysis	Source
Infection prevalence in the hospital	0.02	Triangular distribution (min = 0, max = 0.5, mode = 0.2)	National Records Scotland,^7^ Public Health Scotland,^22^Scottish government^31^(estimated)
Infection prevalence in the community	0.05	Triangular distribution (min = 0, max = 0.2, mode = 0.05)	Perez-Reche and Strachan^21^
The probability that an infected resident dies (age-specific)	Drawn for each individual resident from empirical distribution by age: 80+ y: 11% 70–79 y: 6.0% 60–69 y: 2.6% 50–59 y: 0.71% 40–49 y: 0.18% 30–49 y: 0.09% 20–29 y: 0.04% 18–20 y: 0.007%	No (This parameter does not impact our main model output, the number of infected residents, significantly.)	Ferguson et al,^13^Kulu and Dorey^32^ The Infection Fatality Rate (IFR) for Scotland is adjusted based on the overall aged-adjusted IFR value for the UK and the relative IFR value (=1.18) for other urban areas in Scotland. Most of the population (>80%) in North Lanarkshire live in areas classified as other urban areas.
The probability that an infected staff member dies	Drawn for each individual staff member from a uniform distribution (0.0003–0.022)	No	Ferguson et al,^13^Kulu and Dorey^32^
The no. of contacts that a resident has with other residents per day	Drawn for each individual resident from a Poisson distribution with a mean of 3.9 contacts per resident per day	Mean of the Poisson distribution is drawn from a triangular distribution (min, 1; max, 5; mode = 3.9)	Van den Dool et al,^11^Chamchod and Ruan^33^
The no. of contacts that a staff has with other staff per day	Drawn for each individual staff member from a Poisson distribution with a mean of 7.3 contacts per staff member per day	Mean of the Poisson distribution is drawn from a triangular distribution (min, 1; max, 10; mode, 7.3)	Van den Dool et al^11^
The no. of contacts that a staff has with residents per day	Drawn for each individual staff member from a Poisson distribution with a mean of 16.2 contacts per staff per day	Mean of the Poisson distribution is drawn from a triangular distribution (min, 10; max, 20; mode, 16.2)	Van den Dool et al,^11^Chamchod and Ruan^33^
The no. of contacts that a staff has with visitors per day	5.0 contacts per staff member per day	Triangular distribution (min, 0; max, 10; mode, 5.0)	Discussions with the manager and staff of the representative care home
The probability that a resident comes into contact with another resident in the other unit	20%	Triangular distribution (min, 0; max, 0.5; mode, 0.2)	Discussions with the manager and staff of the representative care home
The average no. of people visiting a resident per day	1.0 visitor per resident per day	Triangular distribution (min, 0; max, 2.0; mode, 1.0)	Van den Dool et al,^11^Port et al^34^
The rate at which residents leave the care home because of deaths caused by other reasons, moving to another facility, admitted to hospitals, or returning to their own home (rare)	0.005 deaths or discharges per resident per day	Triangular distribution (min, 0.001; max, 0.005; mode, 0.004)	Scotland Information Services^19^ (Calculated from data for care homes in North Lanarkshire)
Staff turnover rate	24% per year	Triangular distribution (min, 0.1; max, 0.5; mod, 0.24)	Scottish Care^35^
The probability that an infected resident will develop symptoms	Drawn for each individual resident from empirical distribution: 80+ y: 0.9 70–79 y: 0.85 60–69 y: 0.8 50–59 y: 0.75 40–49 y: 0.7 30–49 y: 0.65 20–29 y: 0.6 18–20 y: 0.55	Triangular distribution (min, 0.5; max, 0.9; mode, 0.8)	Ferguson et al,^13^Verity et al^36^
The probability that an infected staff member will develop symptoms	0.7	Triangular distribution (min, 0.5; max, 0.9; mode, 0.7)	Ferguson et al,^13^Verity et al^36^ (for a population like the United Kingdom or the United States)
The probability that a symptomatic resident has severe symptoms	Drawn for each individual resident from empirical distribution: 80+ y: 0.28 70–79 y: 0.25 60–69 y: 0.17 50–59 y: 0.11 40–49 y: 0.05 30–49 y: 0.03 20–29 y: 0.01 18–20 y: 0.001	No (This parameter does not affect no. of infections significantly given the assumptions that symptomatic individuals are isolated)	Ferguson et al,^13^Kulu and Dorey^32^ The proportion of symptomatic cases requiring hospitalizations for Scotland is adjusted based on the overall aged-adjusted value for the United Kingdom
The probability that a symptomatic staff member has severe symptoms	Drawn for each individual staff member from a uniform distribution (0.01–0.17)	No	Ferguson et al,^13^Kulu and Dorey^32^
The probability that an individual (resident or staff) is infected after coming into contact with another infectious individual (resident, staff or visitor)	0.02	Triangular distribution (min, 0.001; max, 0.1; mode, 0.02)	Ferguson et al,^13^Wang et al,^37^Tang et al^38^
The time elapsed between first exposure and becoming infectious	Lognormal (μ = 1.16, σ = 0.85)	No (This parameter does not significantly affect number of infections as exposed individuals are not infectious. Also, values for this parameter are relatively consistent across studies.)	Lauer et al,^39^McAloon et al,^40^Nishiura et al^41^ (log normal mean, 4.6; SD, 4.8)
The time elapsed between becoming infectious and onset of symptoms	Discrete uniform distribution (1,3)	No (Values for this parameter are consistent across studies.)	He et al,^42^ Gatto et al,^43^ Byrne et al^44^
The time elapsed between onset of symptoms and recovery (or recovery time for those who remain asymptomatic)	Asymptomatic: log normal (μ = 2.049, σ = 0.246) Symptomatic: Mild: log normal (μ = 2.049, σ = 0.246) Severe: log normal (μ = 2.624, σ = 0.170)	No (There is a strong consensus about the distribution of this parameter in literature.)	Kerr et al,^17^ Wölfel et al^45^
The reduction of resident–resident and staff–staff interactions when social distancing is implemented	0.75	Triangular distribution (min, 0.2; max, 0.9; mode, 0.75)	Assumed (based on other models’ assumption^13,46^ and discussions with care home staff and managers)
The sensitivity of RT-PCR test	0.7	Triangular distribution (min, 0.6; max, 0.98; mode, 0.7)	Watson et al,^47^ Arevalo-Rodriguez et al^48^
The lag between testing and test result	1 day	No (implemented in scenario-based uncertainty analysis)	Discussion with representatives from Public Health Medicine (NHS Lanarkshire) and Lanarkshire Health and Social Care Partnership
Effectiveness of isolation of infected residents	100%	50%, 75%, and 100%	Assumed (based on other models’ assumptions^13,46^)

### Intervention scenarios

We considered the impact of 9 different intervention strategies summarized in Table 2. The reference intervention strategy (Inter1) was based on discussions with local care-home stakeholders in Lanarkshire and aligned with the guidance from the Scottish government for controlling SARS-CoV-2. Interventions such as hand hygiene and using personal protective equipment (PPE) change the infection probability per contact, representing the reduction in transmission risk and an increase in compliance. Residents and staff members who are symptomatic or tested positive are isolated and excluded from work respectively the day after being tested because we assumed it would take 1 day for results to be returned in base-case simulations. Because standard RT-PCR testing is highly specific,^20^ we assumed perfect specificity. The COVID-19 epidemic in the general population was assumed to be ongoing at a constant prevalence within the simulated time (1 year) because we focused on interventions that do not shield the care home from the external world. Finally, the intervention strategies we examined were in force during this period.

**Table 2.** Summary of Intervention Strategies Considered

Intervention Strategy	Description
Inter0	No intervention
Inter1	Isolation of symptomatic cases & testing of new admissions (2 tests) and social distancing and restricted visiting (referred to as the reference intervention).
Inter2	Inter1 and 14-d compulsory isolation for new admissions regardless of the result of their tests
Inter3	Inter1 and adaptive testing (ie, testing staff and residents and the care home is closed to new admissions when there is a symptomatic case, reopening when all symptomatic and confirmed residents recover)
Inter4	Inter3 and 14-d compulsory isolation for new admissions
Inter5	Inter1 and weekly testing of residents
Inter6	Inter1 and weekly testing of staff
Inter7	Inter1 and weekly testing of staff and residents
Inter8	Inter6 and 14-d compulsory isolation for new admissions
Inter9	Inter7 and 14-d compulsory isolation for new admissions

### Outcomes

A stochastic ABM produces different outputs for the same parameter set; therefore, it requires a large number of simulations to gain an understanding of the behavior of the system over time. We ran 300 simulations for each scenario because the mean outputs converged after this number of simulations. The outcomes we considered in the results were the prevalence of infected residents over time (means and distribution of prevalence at peak) and the cumulative number of infected residents (means, medians, interquartile ranges [IQRs], and 1.5×IQRs).

### Uncertainty and sensitivity analyses

We performed global probabilistic sensitivity analyses for parameter uncertainty for the reference intervention (Inter1) and weekly testing of staff strategies (Inter6). The probability distributions of the analyzed parameters are summarized in Table 1. We adopted the Latin hypercube sampling (LHS) method to generate 300 sets of samples, and we performed 100 iterations for each set (ie, 30,000 simulations in total). We calculated the partial rank correlation coefficient (PRCC) to determine the strength of the relationship between each LHS parameter and each outcome measure. We also examined how robust the relative effectiveness of interventions were with respect to the most impactful uncertain parameters determined in the PRCC analysis.

We assessed the impact of the testing interval between 1 and 30 days on the effectiveness of routine testing interventions (Inter6, Inter7, Inter8, and Inter9). We also tested the robustness of the findings to the care home’s capacity, structure, and staff pooling system (Supplementary Table S4-1 in Appendix S4 online). Finally, we examined model outputs with infection prevalence in the community set to historical daily data in Scotland adjusted for undetected cases and start the simulation without a seeded infection.^21,22^

### Statistical analysis

We used the Welch *t* test at a significant level of α = 0.05 to perform hypothesis testing for difference in the mean cumulative numbers of infections after 90 and 180 days between scenarios (Appendix S2 online). We also adopted the Bonferroni correction method in which the *P* values were multiplied by the number of tests to counteract the potential type 1 error in multiple comparisons.

## Results

### Spread of COVID-19

**Fig. 2.**
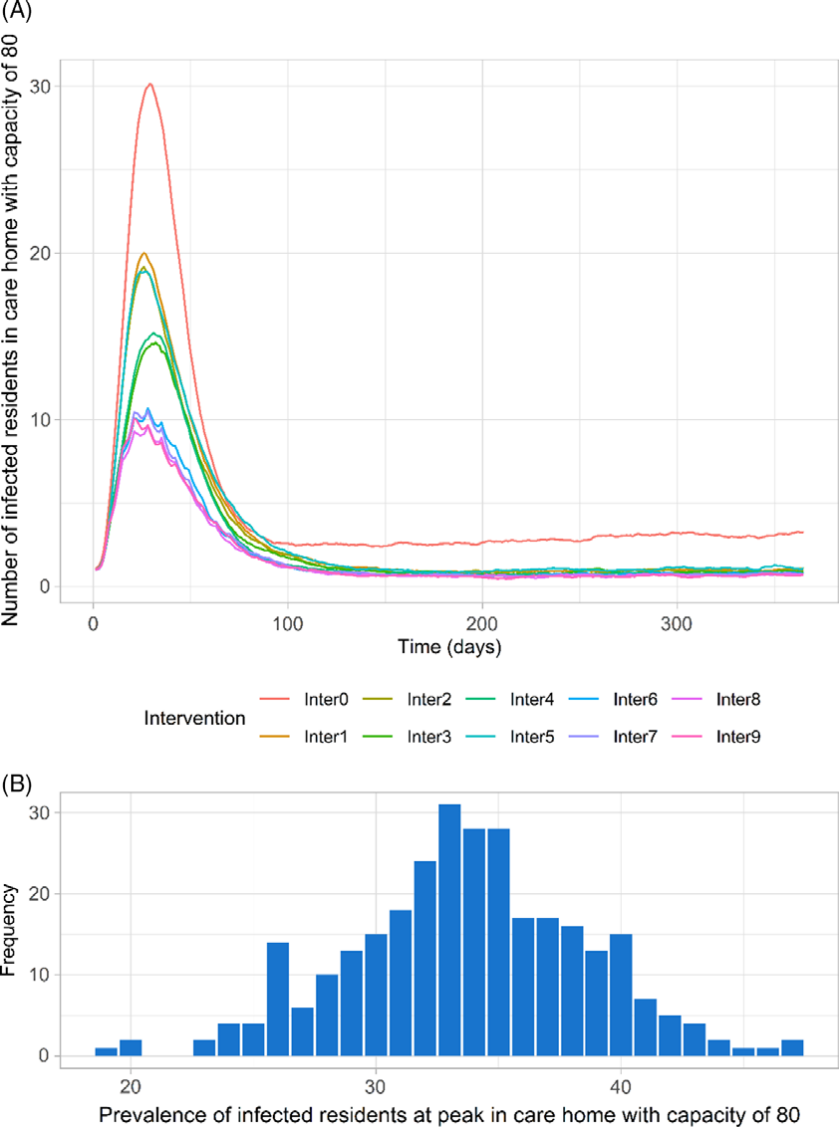
Time series of COVID-19 prevalence among residents in care home with capacity of 80 residents across all scenarios using the base case parameters (means of 300 simulations for each scenario) (A) and distribution of the prevalence at peak for no intervention scenario (Inter0) using the base case parameters for 300 simulations (B). (**Inter0:** No intervention; **Inter1:** Reference intervention (isolation of symptomatic/confirmed residents, testing of new admissions, closed to visitors, social distancing); **Inter2:** Inter 1 + isolation upon admission; **Inter3:** Inter1 + adaptive testing strategy; **Inter4:** Inter3 + isolation upon admission; **Inter5:** Inter1 + Weekly testing for residents; **Inter6:** Inter1 + weekly testing for staff; **Inter7:** Inter1 + weekly testing for staff and residents; **Inter8:** Inter6 + isolation upon admission; **Inter9:** Inter7 + isolation upon admission).

In all scenarios, the mean prevalence of infected residents peaked ~30 days after the first infection in the care home, decreased, then stabilized after around ~90 days (Fig. 2A). The distribution of prevalence at peak (mean, 34; standard deviation [SD], 4.9; range, 19–47) in the no intervention scenario is illustrated in Figure 2B. Relatively large variations in prevalence values are due to stochastic uncertainty of interactions within the care home and disease progression.

**Fig. 3.**
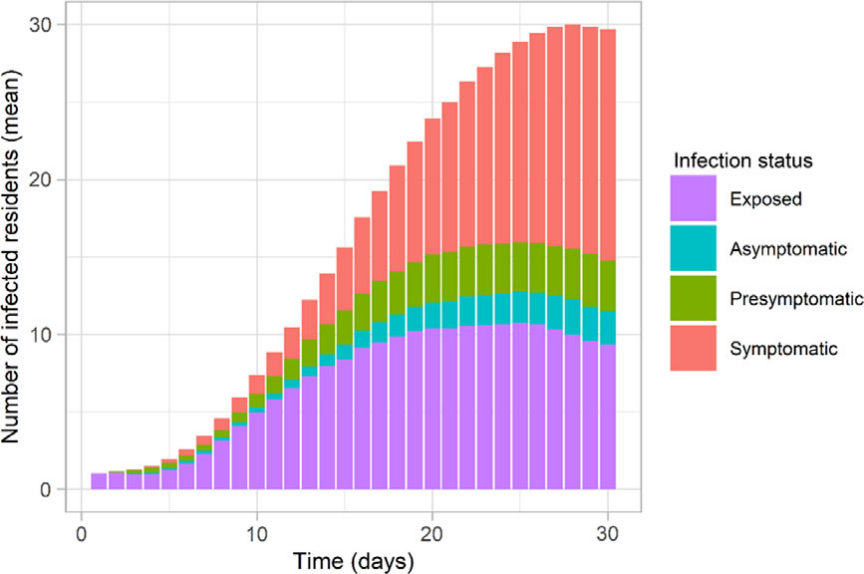
Time series of prevalence of infected residents (mean) in different infection status across 300 simulations when no intervention is implemented (Inter0) using the base case parameters.

In the absence of any control measures and spontaneous changes in the behaviors of individuals, the introduction of a single infected resident resulted in an outbreak (ie, at least 2 residents are infected) in 99.7% of simulations (299 of 300); in 1 simulation, transmission died out quickly. By the time that any infected residents manifested COVID-19 symptoms, an average of 6 residents (SD, 4.2; range, 1–23) had acquired the infection but may not (yet) have shown symptoms. Infected cases that did not (yet) display symptoms made up approximately half of all infections among residents (Fig. 3), which aligns with reported data.^23^ Additionally, the proportion of asymptomatic cases among infected residents in our study (7%; range, 4%–10%) shows a good approximation of observed data for long-term aged care (8%; range, 3%–18%).^24-26^

### Effectiveness of the examined intervention strategies

**Fig. 4.**
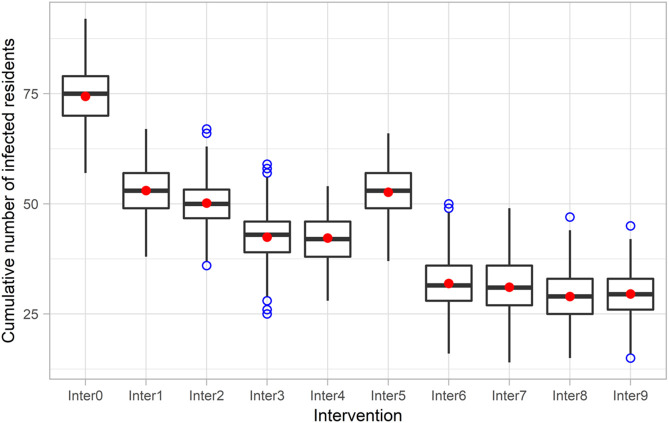
Box plot of cumulative numbers of infected residents 90 days after a resident is infected at the start of the simulation in nine intervention scenarios using the base case parameters (The result is presented as a box plot − lower hinge: 25% quantile; lower whisker: smallest observation greater than or equal to lower hinge − 1.5×IQR; middle: median; upper hinge: 75% quantile; upper whisker: largest observation less than or equal to upper hinge + 1.5×IQR; red dot: mean; blue dot: outlier). Note. IQR, interquartile range.

Implementing the reference intervention, which combines isolation of symptomatic residents, testing of new admissions, social distancing, and restricted visiting (Inter1), clearly lowered the peak and reduced the cumulative number of infections after 90 days compared to the no intervention scenario (Inter0) (Fig. 2A and Fig. 4). There was very strong evidence (*P* < .001) for rejecting the null hypothesis in favor of the alternative hypothesis that the mean cumulative number of infected residents for the reference intervention scenario was lower than the mean when doing nothing (95% CI of the difference, 20.4–22.4 [90 days]).

Adding the 14-day compulsory isolation of new admissions (Inter2) slightly decreased the number of infections compared to the reference intervention strategy (*P* < .001; 95% CI of the difference, 1.9–3.7 [90 days]). Replacing the isolation of new admissions in strategy Inter3 with or adding the adaptive testing intervention (Inter4) further improved the outcomes. However, because the care home was closed to new admissions for part of the time in these scenarios, the total number of residents were smaller than those in other scenarios, contributing to the lower infections shown in Figure 4 for interventions Inter3 and Inter4. Furthermore, weekly resident testing (Inter5) did not lead to lower infections when compared with the reference intervention (Inter1) (*P* ~ 1.0).

Weekly testing of staff in the presence of the reference intervention strategy (Inter6) was more effective than Inter2–Inter5, significantly reducing the peak and the cumulative number of infected residents. This intervention strategy reduced the cumulative infections among residents by ~20 cases after 90 days compared to the reference intervention (Inter1) and by ~10 cases compared to adaptive testing (Inter4) (*P* < .001). A more stringent strategy that involves routine testing for both residents and staff (Inter7) showed little evidence in improving the outcomes (*P* ~ 1.0; 95% CI of the difference: –0.1 to 1.8 [90 days]). Supplementing these routine testing interventions with isolation of new admissions (Inter8 and Inter9) only slightly reduced the peak and cumulative outcomes. Additional plots of modeling results for different time intervals are included in Appendix S3 (online).

### Effectiveness of various routine testing strategies and compliance

**Fig. 5.**
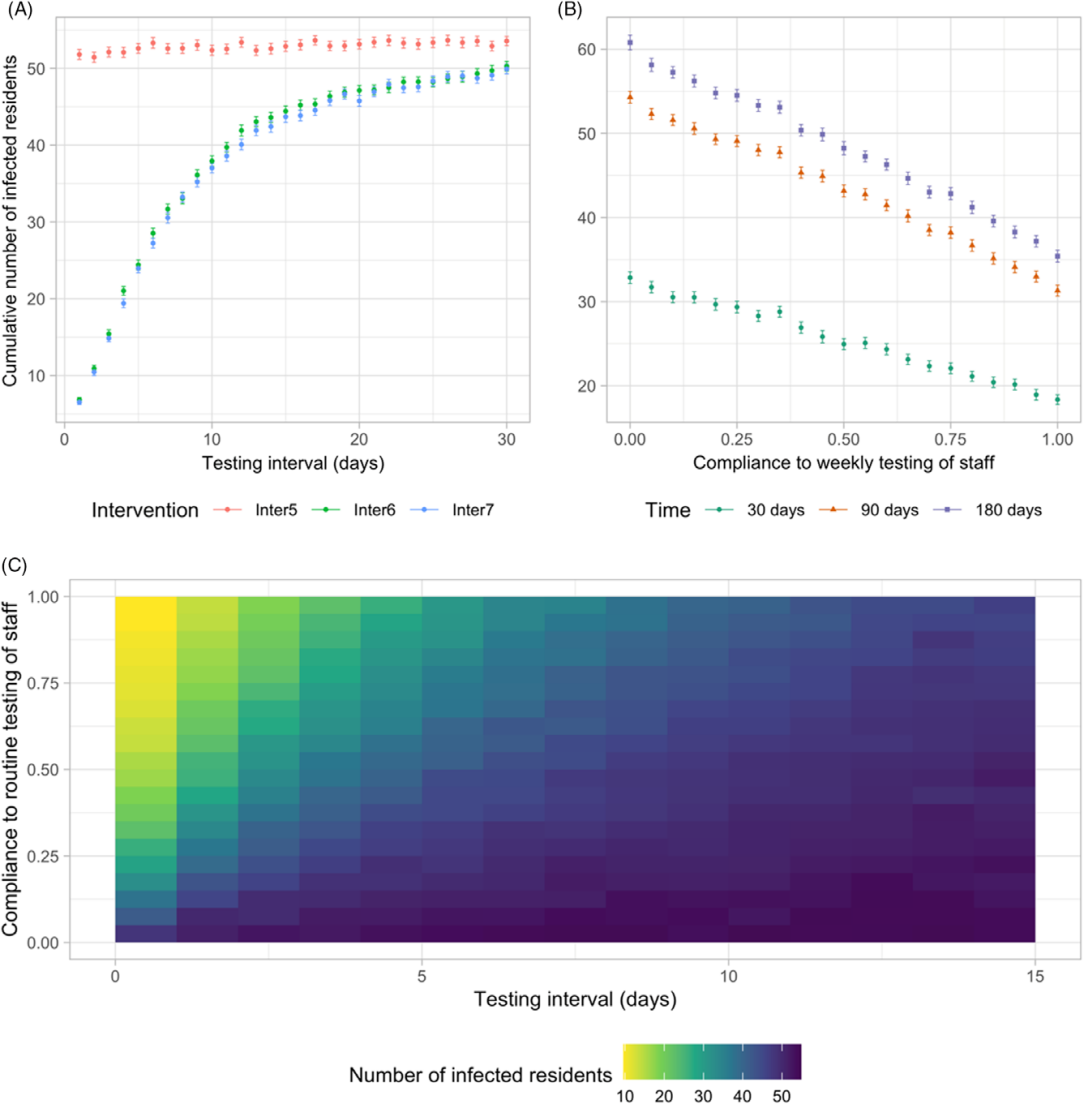
Effectiveness of different routine testing strategies and compliance. (A) The impact of different testing intervals in routine testing scenarios on the cumulative number of infections after 90 days. (B) The impact of compliance to weekly testing of staff (Inter6) on the cumulative number of infections after 30, 90, and 180 days. Other parameters take the values at base case. (Dots denote the mean values of 300 simulations and error bars represent 95% confidence interval of the mean.) (C) Heatmap plot for the impact of testing interval and compliance to routine testing of staff (Inter6) on the cumulative number of infections after 90 days.

Routine testing of residents (Inter5) was predicted to be no more effective than the reference intervention strategy (Inter1) regardless of testing frequency (*P* ~ 1.0; 95% CI of the difference, –0.1 to 2.1 [180 days]). The effectiveness of routine testing of staff (Inter6) and of staff and residents (Inter7) decreased nonlinearly with increased testing intervals (Fig. 5A). The difference between the 2 interventions (Inter6 and Inter7) reduced as the infection probability reduced (Fig. S4-3 online). Increasing compliance to routine testing of staff linearly reduced the cumulative number of infected residents (Fig. 5B). Moreover, compliance with routine testing of staff had a significant effect on the model outcome when a testing interval was <10 days (Fig. 5C).

### Sensitivity and uncertainty analyses

Outputs from the PRCC analyses are summarized in Appendix S4 (online). The PRCC values measure the associations between each of the parameters and the cumulative number of infected residents after 90 days. The infection probability per contact, the infection prevalence in the community, and the average staff–resident contact rate were the highest contributors to uncertainty in the cumulative prevalence of COVID-19 in care homes in both scenarios. Additionally, the outcomes in testing scenarios were sensitive to test sensitivity. Increasing these parameters led to an increase in cumulative COVID-19 prevalence in care homes. Due to the large correlation between the infection probability and the outcomes, measures including individuals’ hand hygiene and PPE that reduce the risk of transmission are highlighted as extremely important for COVID-19 prevalence.

The examined outcomes were also sensitive to the infection prevalence in the community and staff–resident contact rate but to a significantly lesser extent. The model outcomes were sensitive to the staff–resident contact rate but not to staff–staff and resident–resident contact rates in both scenarios. The difference in sensitivity to different types of contacts ocurred because a social distancing measure was implemented in the reference intervention, and we assumed that the intervention reduced staff–staff and resident–resident contact while staff–resident contact rates remained unchanged. Test sensitivity affected the effectiveness of routine testing of staff strategy. The model outcomes were not sensitive to the number of visitors allowed. Across values of the most impactful parameters, the relative effectiveness of intervention strategies remained unchanged (Supplementary Fig. S4-3 in Appendix 4 online).

The findings regarding the relative effectiveness of interventions were robust when modifying the structures (unit size and residents-per-staff ratio) and capacity of the care home. Unit size or residents-per-staff ratio did not significantly impact the cumulative number of infected residents. Neither did care-home capacity affect the proportion of infections among residents. Furthermore, when we used the daily data of Scotland adjusted for undetected cases^21,22^ and started the simulation without a seeded infection, the order of the impact of strategies remained robust.

## Discussion

We present an ABM that captures heterogeneity and stochasticity of individuals’ disease progression and interaction patterns and their effect on transmission dynamics of SARS-CoV-2 and the effectiveness of control interventions in a care-home setting. Care homes are diverse in terms of their resident population, structure and management, and ABMs have more flexibility compared to simpler epidemiological compartment models to reflect this variation and to examine how it influences findings. The stochastic feature of ABM is well-suited for simulating a small population in an intricate setting like a care home, where chance events can lead to major effects. Furthermore, while deterministic compartment models yield a single output for each parameter set, an ABM produces a distribution of outputs accounting for stochastic uncertainty of interactions within the care home and disease progression.

Our simulations show that once SARS-CoV-2 is introduced into care homes, it spreads very quickly and stopping the spread is very difficult. Because risk of transmission per contact appears to be the most impactful factor on the prevalence and cumulative prevalence of infections among residents, interventions such as hand hygiene and PPE that reduce this risk are crucial for controlling the spread of SARS-CoV-2. The importance of these measures in controlling COVID-19 should be emphasized and reinforced among staff in care homes as they may become less compliant when community transmission improves and interventions are relaxed.

Among the examined SARS-CoV-2 testing strategies, routine testing of staff appears to be the most effective and practical approach in the presence of the reference intervention strategy. When the risk of transmission per contact is reduced by enhancing compliance to hand hygiene and PPE use, the strategy of routine testing of staff is as effective as more stringent interventions strategies. This includes the combination of this strategy and 14-day compulsory isolation of new admissions, routine testing of both staff and residents with/without isolation of new admissions. Routine testing of residents does not show additional effect compared to the reference intervention strategy. Therefore, our model predictions suggest that routine testing should target staff in care homes in conjunction with encouragement and support to enhance compliance to hand hygiene and using PPE.

Our modeling results on the effectiveness of routine testing of staff and residents are supported by a number of recently published studies. Weekly universal testing of all staff and residents irrespective of symptoms conducted in 123 West Virginia nursing homes showed that this intervention was more effective in lowering the prevalence of COVID-19 than daily symptom-based resident and staff screening.^27^ Other empirical studies in nursing homes in the United States and France also reported that routine universal testing helped identify cases among staff and residents more quickly and interrupted transmission in the facility.^28-30^ These studies, however, did not examine the impact of routine testing targeting staff only and compared it to resident testing, which is easier to study in a simulation model such as ours than designing a controlled experiment.

Regarding testing intervals, our model predictions, along with discussions with local experts and management regarding feasibility, suggest that routine testing of staff should be carried out every 7–10 days. Although more frequent intervals of testing of staff result in better outcomes, this may not be feasible and is costly. The adverse effects of more frequent testing of staff include increased workload, time pressure, worsened staff shortages, and decreasing tolerance; therefore, they may lead to reduced compliance to testing among staff members. Increasing workload and time pressure may, in turn, affect other care activities provided to residents and staffs’ compliance with testing and hand hygiene, which has greatest impact on the transmission of SARS-CoV-2. A more frequent testing policy could be tailored to care homes with outbreaks to achieve the best outcomes at an acceptable cost. At this stage, we did not explicitly consider the implications of these additional costs in our model.

Care homes are interested in developing strategies for visitors, which is important for the welfare of residents. The model outcomes are not sensitive to the number of visitors allowed because staff that interact with residents more frequently also provide an entry point for infection. This finding suggests that relaxing the visiting policy, will not significantly impact the effectiveness of the examined interventions. However, the assumption that the model starts with a seeded infection limits this interpretation. Further work is required to combine a model such as the one we present with a model of prevalence in the community over time to explore the effect of relaxing visiting under different scenarios of infection prevalence in the community.

This work has a number of limitations. First, we assumed that the level of COVID-19 in the community is static. However, varying this parameter or using time series data of Scotland did not affect the intervention ranking. Second, the model does not account for the potential that residents with atypical symptoms are not successfully detected and, therefore, isolated in a timely manner. However, we conducted sensitivity analysis to consider varying values of the probability residents develop symptoms to reflect this uncertainty because we modeled atypical symptomatic residents who are not detected as asymptomatic residents. We found that this parameter does not affect the outcomes. Third, although we carried out uncertainty and sensitivity analyses on a care home’s size and structure, the diversity of this setting in terms of characteristics of resident populations, health and care services provided, and management would limit the generalization of our findings. Finally, we have not evaluated the cost-effectiveness of the examined interventions which would impact the feasibility of implementation.

In conclusion, our analysis sheds light on the transmission dynamics of COVID-19 in care homes. The effectiveness evaluation of different infection control intervention strategies has potentially significant implications for public health policy making. Infection control interventions in care homes need to be both effective in containing the spread of COVID-19 and also feasible to implement in this setting which has a dual nature: a healthcare institution and a home. Routine testing that targets staff is most effective and practical, and more rigorous testing strategies may not induce additional impact. We also emphasize the importance of interventions such as hand hygiene and using PPE that reduce risk of transmission in inter-individual contacts on the spread of SARS-CoV-2 and the COVID-19 pandemic.

## Supplementary material

For supplementary material accompanying this paper visit https://doi.org/10.1017/ice.2020.1369. click here to view supplementary material
