# Evaluating intervention strategies in controlling coronavirus disease 2019 (COVID-19) spread in care homes: An agent-based model – CORRIGENDUM

**DOI:** 10.1017/ice.2021.36

**Published:** 2022-05

**Authors:** Le Khanh Ngan Nguyen, Susan Howick, Dennis McLafferty, Gillian H. Anderson, Sahaya J. Pravinkumar, Robert Van Der Meer, Itamar Megiddo

In the above article^1^, the author names should appear as follows:

Le Khanh Ngan Nguyen, Susan Howick, Dennis McLafferty, Gillian H. Anderson, Sahaya J. Pravinkumar, Robert Van Der Meer and Itamar Megiddo
