# Supplementary material for: Evaluating intervention strategies in controlling coronavirus disease 2019 (COVID-19) spread in care homes: An agent-based model
Source: Infect Control Hosp Epidemiol. 2020 Dec 14:1–11. doi: 10.1017/ice.2020.1369 (PMC7783094; doi:10.1017/ice.2020.1369)
Supplement: Supplementary file 1 [file S0899823X20013690sup.zip › S0899823x20013690sup001.docx]

**SUPPLEMENTARY MATERIALS**

**APPENDIX S1: THE ODD PROTOCOL**

### Purpose and Patterns

The purpose of the model is to understand the spread of COVID-19 and anticipate the prevalence and cumulative number of infected residents over time in a care home. It also aims to examine the effectiveness of various infection control strategies in controlling COVID-19 in this setting. We evaluate our model by its ability to reproduce the patterns of the dynamics of outbreaks reflecting what has happened in Scottish care homes that have experienced outbreaks and the proportion of asymptomatic infections reflecting what has been reported in long term aged care setting in literature.

### Entities, State Variables, and Scales

The following entities are included in the model: two types of agents, namely resident and staff agents, representing residents and staff in the care home. Each agent entity is characterized by a unique set of state variables which is described in greater detail in Table S1-1.

The resident agents are split into two units with own group of staff agents representing care staff members including nurses and nursing/care assistants. There is a separate group of staff agents which are shared between the two units and represent staff in other roles including housekeepers and wellbeing coordinators.

The model runs at a daily time step as epidemiological data are collected on a daily basis and the unit of time commonly used to describe clinical characteristics of COVID-19 in the literature is day. Simulations are 365-day time steps long as this covers the period since the beginning of the pandemic until now and the upcoming period for which the clients are planning.

Table S1-1: The state variables of resident and staff agents and the global environment

| Variable name | Variable type, units and range | Meaning and rationale |
| --- | --- | --- |
| *Resident-agent-specific state variables* | | |
| unitID | Integer, static; no unit; [1,2,3] | The ID of the unit where a resident stays. It affects with whom a resident can come into contact. E.g. A resident only comes into contacts with other residents living in the same unit. |
| Age | Integer, static; years old; [18 – 110] | The age of a resident which affects the infection fatality rate |
| ResidentInState | String, dynamic; no unit; “susceptible”, “exposed”, “asymptomatic”, “presymptomatic”, “symptomatic”, or “recovered” | The state of infection of a resident. Asymptomatic, presymptomatic and symptomatic residents are infectious. |
| Severity | Integer, dynamic; no unit; 0 = no symptom, 1 = mild, 2 =severe | The severity of symptoms in symptomatic cases that affect the duration of infectiousness |
| AdmissionScheduled | Boolean, dynamic; no unit; true/false | This variable denotes a resident agent leaving the care home or dying, and waiting for admission as a new agent |
| Isolation | Boolean, dynamic; no unit; true/false | This variable indicates whether a resident is isolated |
| Tested | Boolean, dynamic; no unit; true/false | This variable indicates whether a resident receives a RT-PCR test for COVID-19 |
| *Staff-agent-specific state variables* | | |
| unitID | Integer, static; no unit; [1, 2, 3] | The ID of the unit where a member of staff works. It affects with whom a staff can come into contact. |
| Employment | String, static; no unit; “casual” (Bank or Agency staff) or “permanent” | The employment status of a staff |
| StaffInState | String; dynamic; no unit; “susceptible”, “exposed”, “asymptomatic”, “presymptomatic”, “symptomatic” or “recovered” | The state of infection of a member of staff |
| Severity_Staff | Integer, dynamic; no unit; 0 = no symptom, 1 = mild, 2 =severe | The severity of symptoms in symptomatic cases that affect the duration of infectiousness |
| AtWork | Boolean; dynamic; no unit; true/false | The variable indicates whether a staff member is on duty |
| SelfIsolation | Boolean; dynamic; no unit; true/false | The variable indicates whether a staff member is self-isolating at home |
| Replaced | Boolean; dynamic; no unit; true/false | The variable indicates whether a staff member is replaced by or replaces another staff member in the next time step |
| Tested | Boolean, dynamic; no unit; true/false | This variable indicates whether a resident receives a RT-PCR test for COVID-19 |

### Process Overview and Scheduling


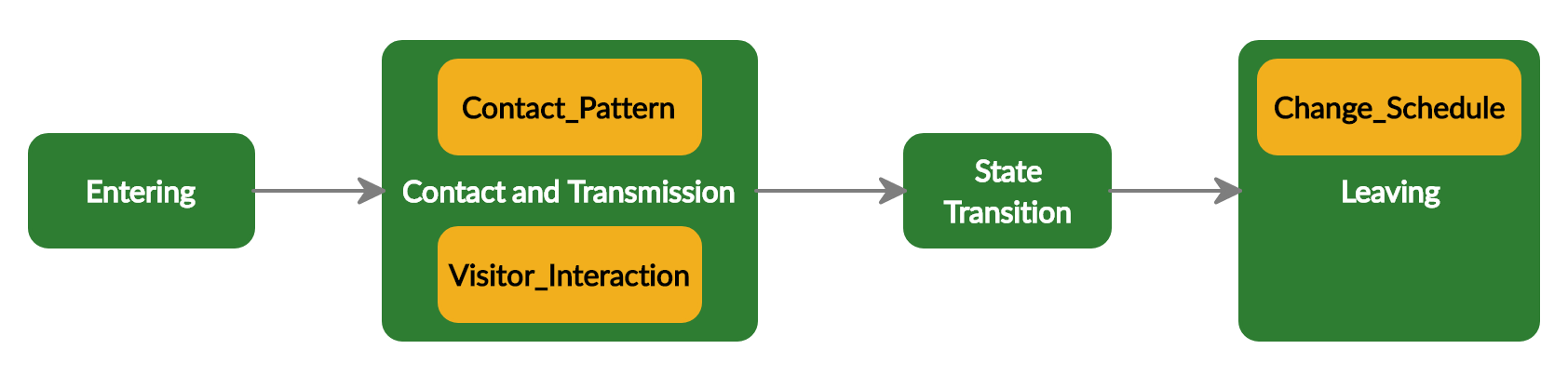


Figure S1-2: The process overview and scheduling of the model at each time step

The model includes five actions executed in the following order at each time step (Figure S1-2):

***Entering:***

*Resident agents:* New residents (AdmissionScheduled is “true”) are admitted. Residents could be admitted from either hospitals or the community at equal probabilities based on discussions with care homes. Their ResidentInState is set to “exposed” at the corresponding probability determined by the parameters InfectionPrevalenceHospital and InfectionPrevalenceCommunity; otherwise, it is set to “susceptible”. AdmissionScheduled is returned the value “false”. The variable Age is drawn from an empirical distribution.

*Staff agents:* If a staff agent’s state variable Replaced is “true”, it is altered to “false”. If its variable AtWork is “false”, it is set to “true” and vice versa. Susceptible permanent staff agents whose state variable AtWork is “true” and casual staff can introduce infections into the care home at the probability defined by the parameter InfectionPrevalenceCommunity. Infected staff agents can either be exposed or infectious (asymptomatic or presymptomatic) at equal probabilities.

***Contact and Transmission:*** Agents (residents and staff agents whose state variable AtWork is “true”) interact with one another following the corresponding contact rates and the rules described in the sub-model *Contact_Pattern*. Transmission occurs at the transmission probability per contact determined by the parameter InfectionProbability when a susceptible agent comes into contact with an infectious agent. When transmission occurs, the infection status of the susceptible agent (i.e. ResidentInState or StaffInState) changes to “exposed”. Interactions between residents and visitors are described in the sub-model *Visitor_Interaction*. Interactions with isolated residents will result in no infection. The order of agents coming into contact with one another is executed randomly within this process.

***State Transition:***

*Exposed 🡪 Pre/Asymptomatic:* Exposed residents and staff agents transit to either the pre-symptomatic state at the probability pSymptomatic and pStaffSymptomatic respectively, or the asymptomatic state at the end of the period determined by the parameter ExposedTime.

*Presymptomatic 🡪 Symptomatic:* Presymptomatic agents develop symptoms (the infection status changes to “symptomatic”) at the end of their pre-symptomatic period defined by the parameter PresymptomaticTime. The probability that a symptomatic agent has severe symptoms is defined by the parameter pSevere and pStaffSevere for resident and staff agents respectively. Symptomatic residents are isolated (Isolation = “true’) without delay. Staff agents who develop symptoms have to self-isolate at home in the next time step until they recover and be covered by another staff member described in the sub-model *Change_Schedule*.

Asymp/Symptomatic 🡪 Recovered: Asymptomatic and symptomatic agents recover at the probability of (1 – DeathProbability) or (1 – StaffDeathProbability) for residents and staff respectively at the end of their Infectiousness duration corresponding to the severity of their symptoms (i.e. asymptomatic, mild or severe symptoms). Their state of infection changes to “recovered”. The variable Isolation of resident agents and SelfIsolation of staff agents are set to “false”. If such staff agents’ state variable Employment is “casual” implying that they have been covered by Bank/Agency staff, it is changed back to “permanent”.

***Leaving:*** The AdmissionScheduled variables of residents who die or leave the care home are set to “true”. These agents represent residents admitted to the care home in the next time step to replace those dying or leaving in this time step. Infected residents die at the end of their infectiousness period at the probability DeathProbability specific to their Age. Residents in other states of infection could die or leave the care home for other reasons at the rate determined by the parameter LeavingRate. Infected staff could die at the probability StaffDeathProbability. Permanent staff could leave the care home for non-covid reasons at a rate defined by the parameter StaffTurnover. Staff who die or leave are replaced by new “susceptible” staff agents with other state variables being set as in Initialization.

### Design Concepts

#### Basic principles:

The model simulates the transmission dynamics of COVID-19 via contacts between individuals including residents, staff, and visitors within a hypothetical care home that represents a Scottish care home. The progression of COVID-19 infection after transmission occurs is described in Figure S1-3 based on the current understanding and evidence of clinical characteristics of COVID-19 ^13,36,42^. It is assumed that recovered people are immune to re-infection in the short term and pre-/asymptomatic individuals are just as likely to transmit infection as symptomatic individual ^49^. Individuals’ characteristics, behaviors and contact network and pattern, along with the operational and managerial features of the care home, can influence how the virus is disseminated. Such information is based on discussions, surveys, and interviews with stakeholders including Social Care, Council, and nursing homes in Lanarkshire. Infections can be imported into the care home by asymptomatic residents upon admission and staff and visitors who acquired the infection somewhere else. Infection control measures are implemented to reduce the imported infections (e.g. testing upon admission, visit restriction) and/or contain the intra-facility transmission by reducing contact rates (e.g. social distancing, isolation) and/or the risk of transmission per contact (e.g. hand hygiene and use of personal protective equipment (PPE)).

####
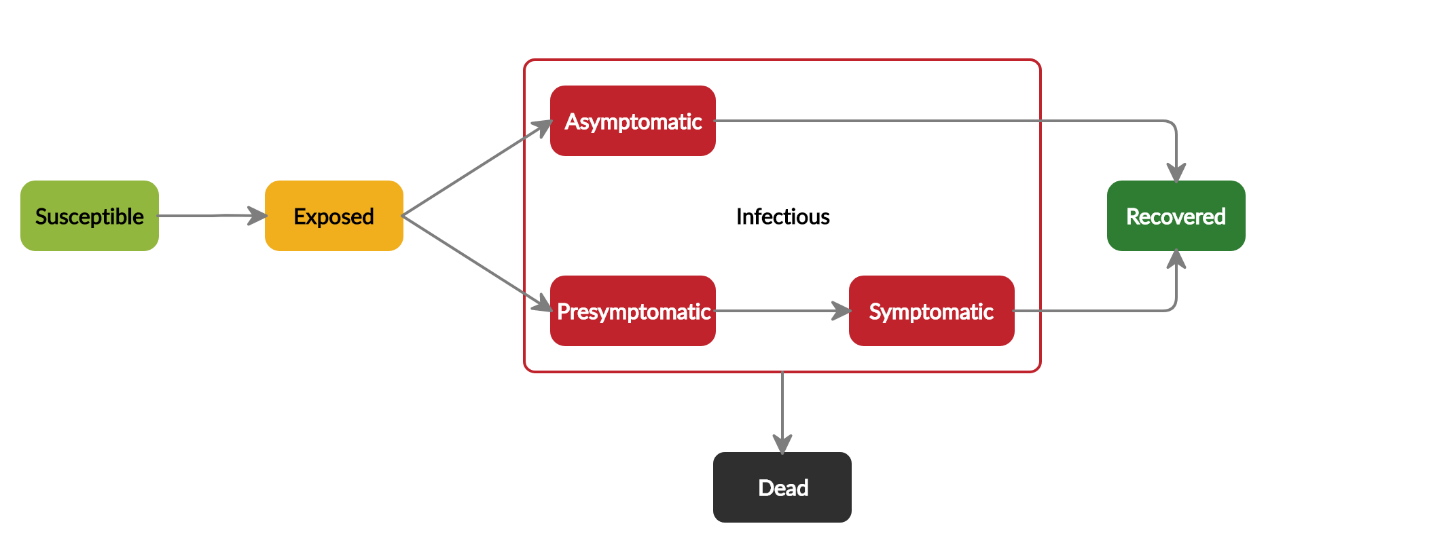
Emergence:

Figure S1-3: The progression of Covid-19 infections

Susceptible people may acquire the infection when exposed to infectious sources. They are infected but not yet infectious (exposed state). Once exposed people become infectious, they can either remain asymptomatic for the entire infectious period or develop symptoms after a pre-symptomatic period. Symptoms could be mild or severe and require hospitalizations. Infectious people will eventually recover or die.

The key outcomes of the model are patterns for the occurrence and recurrence of outbreaks, surges of COVID-19 related deaths and staffs acquiring infections in the care home. These outcomes emerge from the contact network and pattern among residents and staff, how infections are imported into the facility, infection control measures implemented, and staff’s compliance to such measures.

#### Adaptation:

Staff agents that exhibit symptoms or tested positive leave the care home. In isolation scenarios, residents who exhibit symptoms or are tested positive are isolated. When social distancing is implemented, staff and residents adapt to the situation by decreasing their contact rate with other staff members and residents respectively. Residents do not come into contact with other residents in the other unit either.

#### Objectives:

The objective measure used by staff agents to decide whether to comply to infection control measures such as hand hygiene, using PPE, practicing social distancing is the existence of an outbreak in the care home. Their adaptive behaviors aim to reduce transmission rates and help contain the outbreak.

#### Learning:

Learning is not implemented.

#### Prediction:

The staff’s adaptive behavior is based on implicit predictions that leaving when exhibiting symptoms and social distancing will reduce the number of contacts which, in turn, limit the spread of infections and increasing compliance to hand hygiene and using PPE will reduce the risk of transmission per contact.

#### Sensing:

Agents can sense with whom they are in contact. Staff agents can sense the infection status of residents who display symptoms and exposed or asymptomatic residents who are tested positive. Staff who develop symptoms can also sense their own state of health and do not return to work the next day.

#### Interaction:

Figure S1-4: Interactions between residents, staff and visitors in a care home

The dashed lines linking individuals denote their possible ways of interaction. Different colours are used for these lines to distinguish different types of interaction: blue – staff-resident interaction; green: resident-resident interaction; red: staff-staff interaction; black: resident-visitor interaction; purple: staff – visitor interactions.


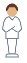

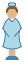

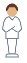

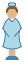

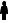


Agents have direct interactions as shown in Figure S1-4. Residents can interact with other residents, staff, and visitors. Staff can interact with other staff and visitors. The network and rates of interactions between residents and staff are defined based on the management policy of a care home and the implemented infection control interventions such as social distancing.

#### Stochasticity:

Residents’ age is initialized stochastically as this characteristic affects residents’ risk of death as an outcome of the infection. Stochasticity is used to describe variability in the parameters that determine the transitions of individuals between different states of infection including the incubation time and the transmission probability. This represents variations in the risk of acquiring the infection and the progression and outcome of the infection among people, influenced by factors such as their health status, underlying conditions, and immune system. Additionally, the interaction between individuals is a stochastic process as randomness exists in contact rates, with whom they come into contact, and the order in which contacts between individuals occur. Another stochastic element is individuals’ compliance to an infection control intervention. Such stochasticity is added to demonstrate how individuals’ heterogenous behaviors, contact network and pattern can affect the spread of the infection. The time at which individuals (staff members, new residents, and visitors) introduce the infection into the care home is also stochastic.

#### ***Collectives***:

The model has three collectives: the two units and the shared ancillary staff group. The collective to which agents belong affects whom they can interact with.

#### Observation:

The primary outputs of interest can be observed via plots of the prevalence of residents in different states of infection, the accumulated number of infected residents over time.

### Initialization

The model is initialized with 80 resident agents and 72 staff agents in the base case. The first unit (UnitID = 1) has 40 resident agents and 33 staff agents. The second unit (UnitID = 2) has 40 resident agents and 32 staff agents. A group of seven staff agents are shared between the two units (UnitID = 3). The number of staff agents present in the care home are 16 and 15 for Unit 1 and 2 respectively. All shared staff are on duty. There are two Bank/Agency staff in Unit 1 and one in Unit 2. The number of residents and staff and the operational structure were provided by the manager of a representative care home for older people. One random resident is exposed to the virus at the beginning of the simulation. The remaining agents are susceptible. The variable Age of residents is drawn from an empirical distribution based on the demographic data of older people adult care homes in North Lanarkshire. Initial values of variables and parameters for the baseline scenario (no intervention) are summarized in Table S1-5. In intervention scenarios, interventions are also scheduled to turn on at particular time steps or under specific conditions (e.g. there is an outbreak in the care home). How relevant state variables and parameters are altered for each intervention is described in greater details in the sub-model *Intervention.*

Table S1-5: Initial values of entities' state variables and parameters

| Variable/ Parameter | Initial value | Sources |
| --- | --- | --- |
| *Resident-agent-specific state variables* | | |
| unitID | 1 for 40 resident agents  2 for the remaining 40 resident agents | Discussions with the manager of a Scottish care home for older people |
| Age | Drawn from empirical distribution:  18-64 years old: 3%  65-74 years old: 13%  75-84 years old: 39%  84-94 years old: 39%  95 and older: 6% | ^19^ (Calculated from data for older people care homes in North Lanarkshire) |
| ResidentInState | “susceptible” with one resident assigned “exposed” |  |
| Severity | 0 |  |
| AdmissionScheduled | False |  |
| Isolation | False |  |
| Tested | False |  |
| *Staff-agent-specific state variables* | | |
| unitID | 1 for 33 staff agents  2 for the other 32 staff agents  3 for the remaining seven staff agents |  |
| Employment | “casual” for two staff from unit 1 and one staff from unit 2  “permanent” for the rest | Discussions with the manager of a Scottish care home for older people |
| StaffInState | “susceptible” |  |
| AtWork | True for all causal staff and 14 permanent staff agents from each unit  False for the rest |  |
| SelfIsolation | False |  |
| Replaced | False |  |
| Tested | False |  |

### Input Data

The model does not use input data to represent time-varying processes in the base case.

### Submodels

Parameters used in the model are described in Table S1-6.

Table S1-6: Parameters used in the model

| Parameter name | Meaning and rationale | Default Value | Sensitivity Analysis | Source |
| --- | --- | --- | --- | --- |
| InfectionPrevalenceHospital | Infection prevalence in the hospital | 0.02 | Triangular distribution (min = 0, max = 0.5, mode = 0.2) | ^7,22,31(estimated)^ |
| InfectionPrevalenceCommunity | Infection prevalence in the community | 0.05 | Triangular distribution (min = 0, max = 0.2, mode = 0.05) | ^21^ |
| Death  Probability | The probability that an infected resident dies (age-specific) | Drawn for each individual resident from empirical distribution:  80+ years old: 11%  70-79 years old: 6.0%  60-69 years old: 2.6%  50-59 years old: 0.71%  40-49 years old: 0.18%  30-49 years old: 0.09%  20-29 years old: 0.04%  18-20: 0.007% | No (This parameter does not impact our main model output, the number of infected residents, significantly. It will likely be the most important parameter when we consider deaths as an outcome of the model) | ^13,32^ (The Infection Fatality Rate (IFR) for Scotland is adjusted based on the overall aged-adjusted IFR value for the UK and the relative IFR value (= 1.18) for other urban areas in Scotland. The majority of population (>80%) in North Lanarkshire live in areas classified as other urban areas.) |
| StaffDeath  Probability | The probability that an infected staff member dies | Drawn for each individual staff member from a uniform distribution (0.0003 – 0.022) | No | ^13,32^ |
| ClosedTo  Admission | The state variable determines whether the care home is opened to admissions | False | No |  |
| ContactRateRR | The number of contacts that a resident has with other residents per day | Drawn for each individual resident from a Poisson distribution with a mean of  3.9 contacts per resident per day | Mean of the Poisson distribution is drawn from a triangular distribution (min = 1, max = 5, mode = 3.9) | ^11,33^ |
| ContactRateSS | The number of contacts that a staff has with other staff per day | Drawn for each individual staff member from a Poisson distribution with a mean of 7.3 contacts per staff member per day | Mean of the Poisson distribution is drawn from a triangular distribution (min = 1, max = 10, mode = 7.3) | ^11^ |
| ContactRateSR | The number of contacts that a staff has with residents per day | Drawn for each individual staff member from a Poisson distribution with a mean of 16.2 contacts per staff per day | Mean of the Poisson distribution is drawn from a triangular distribution (min = 10, max = 20, mode = 16.2) | ^11,33^ |
| ContactRateSV | The number of contacts that a staff has with visitors per day | 5.0 contacts per staff member per day | Triangular distribution (min = 0, max = 10, mode = 5.0) | Discussions with the manager and staff of a Scottish care home for older people |
| ContactAcross  Units | The probability that a resident comes into contact with another resident in the other unit | 20% | Triangular distribution (min=0, max = 0.5, mode =0.2) | Discussions with the manager and staff of a Scottish care home for older people |
| VisitorsPerDay | The average number of people visiting a resident per day | 1.0 visitor per resident per day | Triangular distribution (min = 0, max = 2.0, mode = 1.0 | ^11,34^ |
| LeavingRate | The rate at which residents leave the care home because of deaths caused by other reasons, moving to another facility, admitted to hospitals, or returning to their own home (rare) | 0.005 deaths or discharges per resident per day | Triangular distribution (min = 0.001, max = 0.005, mode = 0.004) | ^19^ (Calculated from data for care homes in North Lanarkshire) |
| StaffTurnover | Staff turnover rate | 24% per year | Triangular distribution (min = 0.1, max = 0.5, mod = 0.24) | ^35^ |
| pSymptomatic | The probability that an infected resident will develop symptoms | Drawn for each individual resident from empirical distribution:  80+ years old: 0.9  70-79 years old: 0.85  60-69 years old: 0.8  50-59 years old: 0.75  40-49 years old: 0.7  30-49 years old: 0.65  20-29 years old: 0.6  18-20: 0.55 | Triangular distribution (min = 0.5, max = 0.9, mode = 0.8) | ^13,36^ |
| pStaff  Symptomatic | The probability that an infected staff member will develop symptoms | 0.7 | Triangular distribution (min=0.5, max=0.9, mode=0.7) | ^13,36^ (For a population like the UK or US) |
| pSevere | The probability that a symptomatic resident has severe symptoms | Drawn for each individual resident from empirical distribution:  80+ years old: 0.28  70-79 years old: 0.25  60-69 years old: 0.17  50-59 years old: 0.11  40-49 years old: 0.05  30-49 years old: 0.03  20-29 years old: 0.01  18-20: 0.001 | No (This parameter does not affect number of infections significantly given the assumptions that symptomatic individuals are isolated with perfect effectiveness) | ^13,32^ The proportion of symptomatic cases requiring hospitalizations for Scotland is adjusted based on the overall aged-adjusted value for the UK |
| pSatffSevere | The probability that a symptomatic staff member has severe symptoms | Drawn for each individual staff member from a uniform distribution (0.01-0.17) | No | ^13,32^ |
| Infection  Probability | The probability that an individual (resident or staff) is infected after coming into contact with another infectious individual (resident, staff or visitor) | 0.02 | Triangular distribution (min = 0.001, max = 0.1, mode = 0.02) | ^13,37,38^ |
| ExposedTime | The time elapsed between first exposure and becoming infectious | Lognormal (μ = 1.16, σ = 0.85) | No (This parameter does not significantly affect number of infections as exposed individuals are not infectious. Also, values for this parameter are relatively consistent across studies.) | ^39-41^ (Lognormal (mean = 4.6, std = 4.8) |
| Presymptomatic  Time | The time elapsed between becoming infectious and onset of symptoms | Discrete uniform distribution (1,3) | No (Values for this parameter are consistent across studies) | ^42-44^ |
| Infectiousness | The time elapsed between onset of symptoms and recovery (or recovery time for those who remain asymptomatic) | Asymptomatic: Lognormal (μ = 2.049, σ = 0.246)  Symptomatic:  -Mild: Lognormal (μ = 2.049, σ = 0.246)  -Severe: Lognormal (μ = 2.624, σ = 0.170) | No (There is a strong consensus about the distribution of this parameter in literature.) | ^17,45^ |
| SDCompliance | The reduction of resident-resident and staff-staff interactions when social distancing is implemented | 0.75 | Triangular distribution (min = 0.2, max = 0.9, mode = 0.75) | Assumed (based on other models’ assumption^13,46^ and discussions with care home staff and managers) |
| TestSensitivity | The sensitivity of RT-PCR test | 0.7 | Triangular distribution (min = 0.6, max = 0.98, mode = 0.7) | ^47,48^ |
| TestDelay | The lag between testing and test result | 1 days | No (Implemented in scenario-based uncertainty analysis) | Discussion with representatives from Public Health Medicine (NHS Lanarkshire) and Lanarkshire Health and Social Care Partnership |
| IsolationEffectiveness | Effectiveness of isolation of infected residents | 100% | 50%, 75%, and 100% | Assumed (based on other models’ assumption^13,46^) |

#### Intervention:

How parameters in the model are modified for each intervention is described in Table S1-7.

Table S1-7: Infection control measures and how model parameters are modified when a measure is adopted

| Infection Control Intervention | | Modified Parameter |
| --- | --- | --- |
| Temporary closure to admissions | Closed to admissions | ClosedToAdmission = true; |
|  | Opened to admissions | ClosedToAdmission = false; |
| Social distancing | | The parameter SDCompliance is used to control the compliance rate to the social distancing measure.  ContactRateRR and ContactRateSS are reduced by (1 –SDCompliance). Residents in different units do not interact with each other (ContactAcrossUnits = 0). |
| Testing upon admission | | Residents can be admitted after having two negative tests.^50^ The probability of identifying true positive after two tests equals to (1 - (1 -TestSensitivity)^2^) |
| Isolation of infected residents | | Their state variable Isolation is set to “true”. Interactions between isolated residents and other individuals result in no infection at the probability *IsolationEffectiveness*. The model assumes no delay between onset of symptoms and isolation. |
| 14-days compulsory isolation of new admissions | | The state variable Isolation of newly admitted residents is set to “true” and scheduled to be revised after 14 days. If they do not either have symptoms or are tested positive, Isolation is changed to “false”. |
| Testing of residents | | Residents who are tested have their state variable Tested set to “true”. After the time delay from testing to test result determined by the parameter TestDelay, the state variable Tested is set to “false” and infected residents are detected at the probability TestSensitivity and isolated. |
| Testing of staff | | Staff members who are tested have their state variable Tested set to “true”. After the time delay from testing to test result determined by the parameter TestDelay, the state variable Tested is set to “false” and infected staff are detected at the probability TestSensitivity and have to self-isolate. |

#### Contact_Patterns:

The patterns for interactions between individuals, following discussions with the manager and care staffs in the care home, are as follow:

- A staff member (UnitID = 1 or 2) can interact with any random staff in the same unit at the rate *ContactRateSS*.
- A staff member (UnitID = 1 or 2) can interact with any random resident in the same unit at the rate *ContactRateRR*.
- A resident can interact with any random resident in the other unit at the probability *ContactAcrossUnits*.
- A staff member in the shared group (UnitID = 3) can interact with any random resident or staff member from any unit at the rate *ContactRateSR* and *ContactRateSS* respectively.
- Isolation of infected residents is assumed to be 100% effective at preventing further transmission (i.e. interactions with isolated residents result in no infection).
- Only staff agents that are present in the care home (the state variable AtWork = true) can interact with other agents.

#### Visitor_Interaction:

Susceptible residents could acquire the infection when coming into contact with visitors who are asymptomatic. Visitors are not explicitly represented as agents in the model as there is no need to consider visitors at an individual level. The model only considers the transmission from visitors to residents. Whether visitors may acquire the infection from residents or staff in the care home and how they spread it elsewhere are not within this model’s scope. In each time step, the number of infectious visitors that come into contact with each susceptible resident are drawn from a Poisson distribution with a mean that equals to (VisitorsPerDay x CommunityProbabilityInfectious). Similarly, the number of infectious visitors that interact with a staff member are drawn from a Poisson distribution with the mean of (ContactRateSV x CommunityProbabilityInfectious). Transmission of such contacts occurs at the probability InfectionProbability.

#### Change_Schedule:

A staff agent who develops symptoms and self-isolates will be covered by another staff member. Its state variable SelfIsolation is set to “true”. The replaced staff agent is randomly chosen from the corresponding staff pool including staff agents that have the same UnitID, are not already on duty (AtWork = false) or in self-isolation (SelfIsolation = false), and have not been scheduled to replace someone else (Replaced = false). The state variables Replaced of both replacing and replaced agents are altered to “true”. If none of staff agents in the pool satisfies these conditions, the agent’ Employment is set to “casual”.

**APPENDIX S2: STATISTICAL ANALYSES**

Table S2-1: Summary of statistics of the cumulative number of infected residents after 90 days in different intervention scenarios

| *Intervention* | *Mean* | *Median* | *Interquartile range* |
| --- | --- | --- | --- |
| Inter0 | 74 | 75 | 70 – 79 |
| Inter1 | 53 | 53 | 49 – 57 |
| Inter2 | 50 | 50 | 47 – 53 |
| Inter3 | 42 | 43 | 39 – 46 |
| Inter4 | 42 | 42 | 38 – 46 |
| Inter5 | 53 | 53 | 49 – 57 |
| Inter6 | 32 | 32 | 28 – 36 |
| Inter7 | 31 | 31 | 27 – 36 |
| Inter8 | 29 | 29 | 25 – 33 |
| Inter9 | 30 | 30 | 26 – 33 |
| Inter5 (every day) | 52 | 52 | 48 – 55 |
| Inter6 (14 days) | 44 | 44 | 40 – 48 |
| Inter6 (20 days) | 47 | 47 | 43 – 51 |
| Inter6 (30 days) | 50 | 50 | 46 – 54 |
| Inter6 (compliance 75%) | 38 | 38 | 34 – 42 |
| Inter6 (compliance 50%) | 43 | 43 | 39 – 48 |
| Inter6 (compliance 25%) | 49 | 49 | 46 – 53 |

Table S2-2: The results of hypothesis testing for the difference in mean cumulative numbers of infected residents after 90 under different intervention strategies using Welch’s t-test (Bonferroni correction)

| *Interventions for hypothesis testing* | | *Welch's t-test results* | | | |
| --- | --- | --- | --- | --- | --- |
|  |  | ***Outcomes after 90 days*** | | ***Outcomes after 180 days*** | |
| *Intervention 1* | ***Intervention 2*** | ***p-value adjusted***  ***(2-tailed)*** | ***95% CI of the difference*** | ***p-value adjusted (2-tailed)*** | ***95% CI of the difference*** |
| Inter0 | Inter1 | < 2.2E-16 | 20.4 , 22.4 | < 2.20E-16 | 27.6 , 30.3 |
| Inter1 | Inter2 | 2.1E-07 | 1.9 , 3.8 | 3.9E-08 | 2.3 , 4.4 |
| Inter2 | Inter3 | < 2.2E-16 | 6.8 , 8.6 | < 2.20E-16 | 7.0 , 9.0 |
| Inter3 | Inter4 | 1.0 | -0.7 , 1.1 | 1.0 | 0.0 , 1.9 |
| Inter1 | Inter5 | 1.0 | -0.5 , 1.3 | 1.0 | -0.9 , 1.4 |
| Inter4 | Inter6 | < 2.2E-16 | 9.4 , 11.2 | < 2.20E-16 | 10.1 , 12.0 |
| Inter1 | Inter6 | < 2.20E-16 | 20.1 , 22.0 | < 2.20E-16 | 22.4 , 24.5 |
| Inter6 | Inter7 | 1.0 | -0.1 , 1.8 | 1.0 | -0.3 , 1.7 |
| Inter6 | Inter8 | 5.4E-05 | 2.0 , 3.9 | 8.1E-07 | 2.1 , 4.1 |
| Inter8 | Inter9 | 1.0 | -1.4 , 0.3 | 1.0 | -1.6 , 0.2 |
| ***Testing intervals*** | | | | | |
| Inter1 | Inter5 (every day) | 1.0 | 0.3 , 2.2 | 1.0 | -0.1 , 2.1 |
| Inter1 | Inter6 (14 days) | < 2.2E-16 | 8.5 , 10.4 | < 2.20E-16 | 10.2 , 12.3 |
| Inter1 | Inter6 (20 days) | < 2.2E-16 | 5.0 , 6.8 | < 2.20E-16 | 6.4 , 8.4 |
| Inter1 | Inter6 (30 days) | 7.9E-07 | 1.8 , 3.7 | 3.8E-12 | 3.0 , 5.1 |
| Inter2 | Inter6 (30 days) | 1.0 | -1.0 , 0.8 | 1.0 | -0.3 , 1.7 |
| ***Compliance to routine testing*** | | | | | |
| Inter1 | Inter6 (compliance 75%) | < 2.2E-16 | 13.8 , 15.8 | < 2.20E-16 | 15.6 , 17.7 |
| Inter1 | Inter6 (compliance 50%) | < 2.2E-16 | 8.9 , 10.9 | < 2.20E-16 | 10.2 , 12.4 |
| Inter1 | Inter6 (compliance 25%) | 1.7E-14 | 3.0 , 4.9 | < 2.20E-16 | 4.0 , 6.0 |


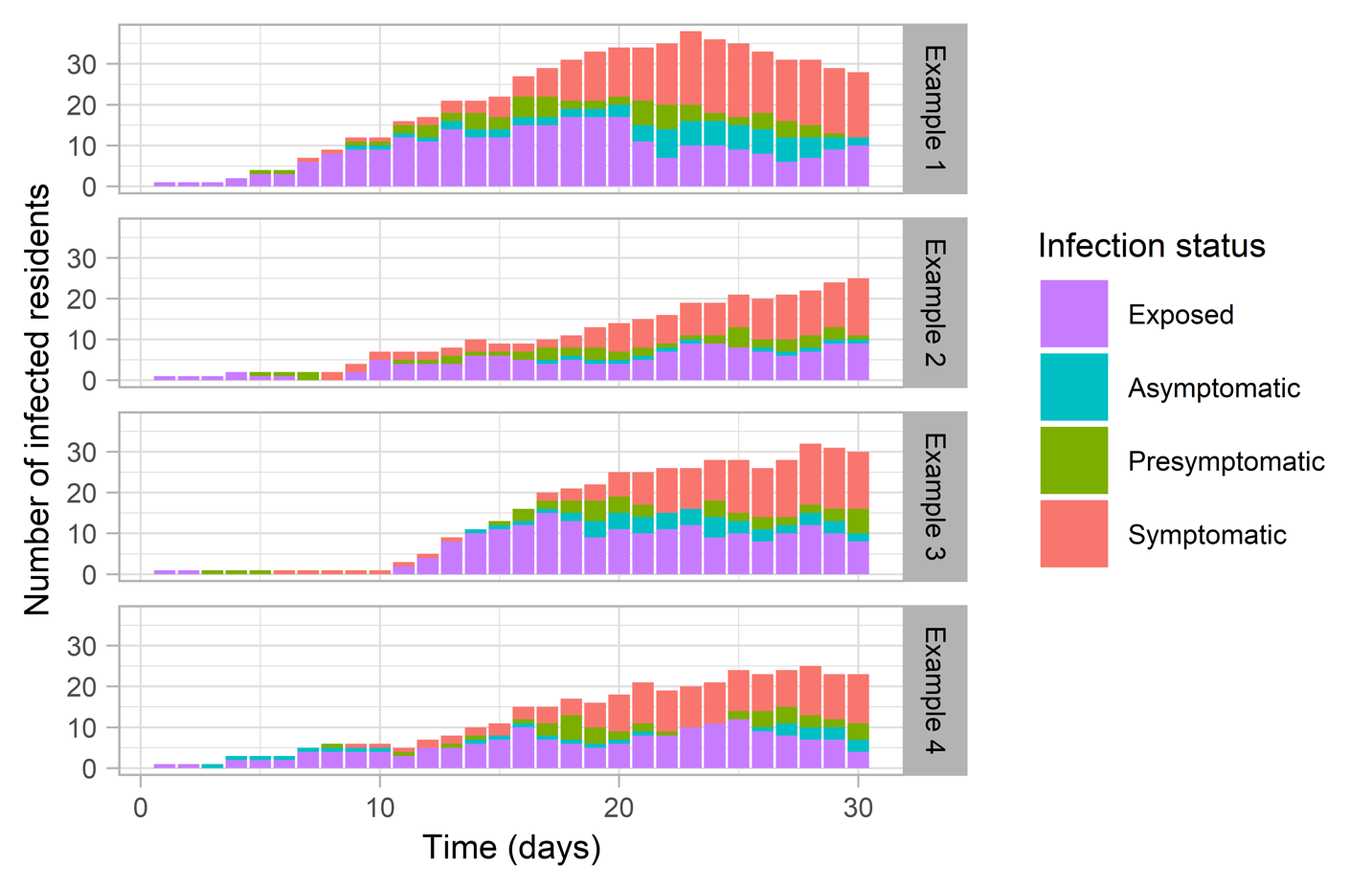
**APPENDIX S3: ADDITIONAL PLOTS OF MODELLING RESULTS**

Figure S3-1: Four simulation examples of prevalence of infected residents in different infection states over time when no intervention is implemented (Inter0) using base case parameters. This figure displays variations of data due to stochastic uncertainty of interactions within the care home and disease progression; parameter uncertainty was not considered in this figure.


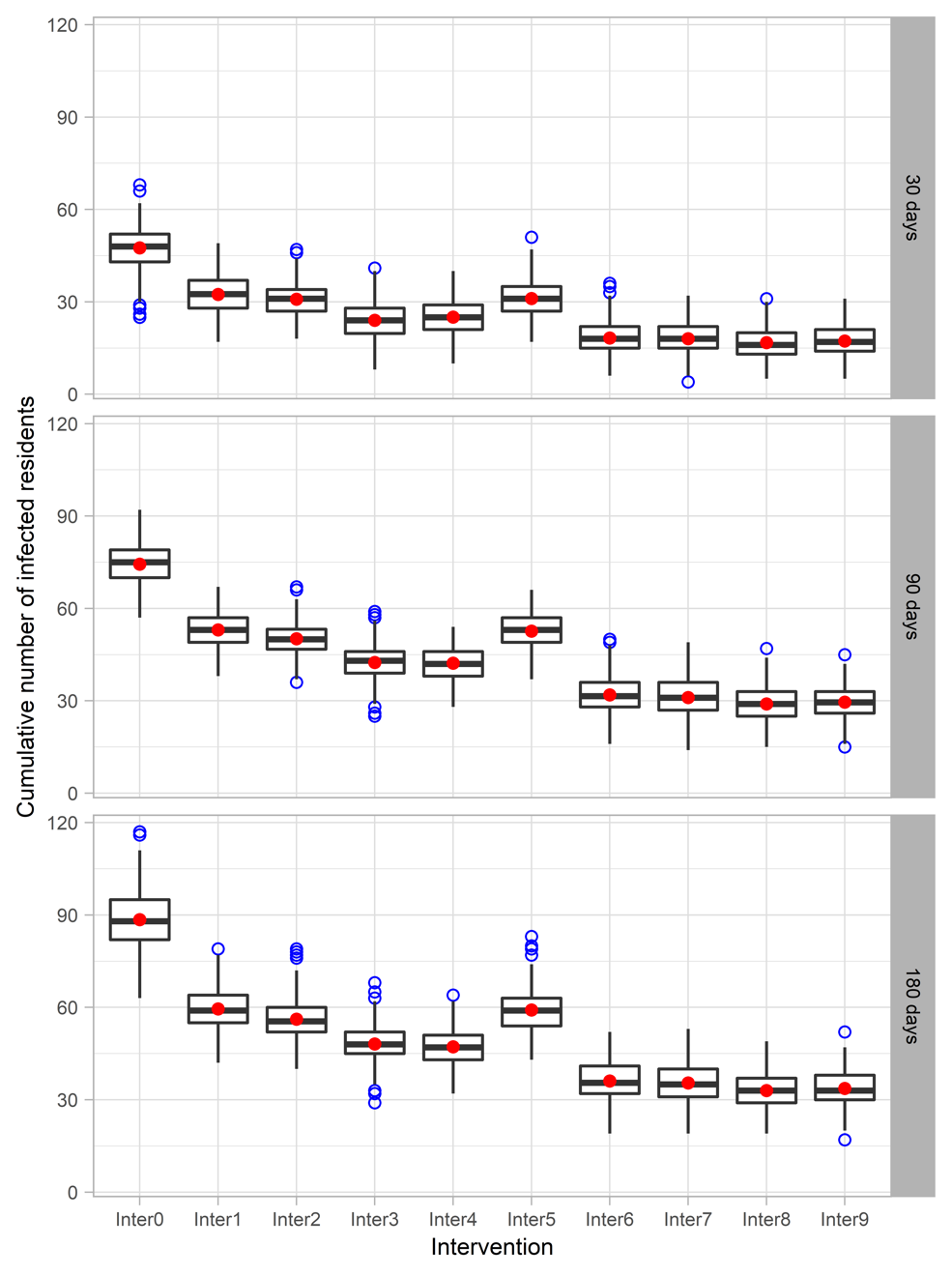


Figure S3-2: Cumulative numbers of infected residents 30, 90, and 180 days in care home with capacity of 80 residents after a resident is infected at the start of the simulation in nine intervention scenarios using the base case parameters

(Inter0: No intervention; Inter1: Reference intervention (isolation of symptomatic/confirmed residents, testing of new admissions, closed to visitors, social distancing); Inter2: Inter 1 + isolation upon admission; Inter3: Inter1 + adaptive testing strategy; Inter4: Inter3 + isolation upon admission; Inter5: Inter1 + Weekly testing for residents; Inter6: Inter1 + weekly testing for staff; Inter7: Inter1 + weekly testing for staff and residents; Inter8: Inter6 + isolation upon admission; Inter9: Inter7 + isolation upon admission)


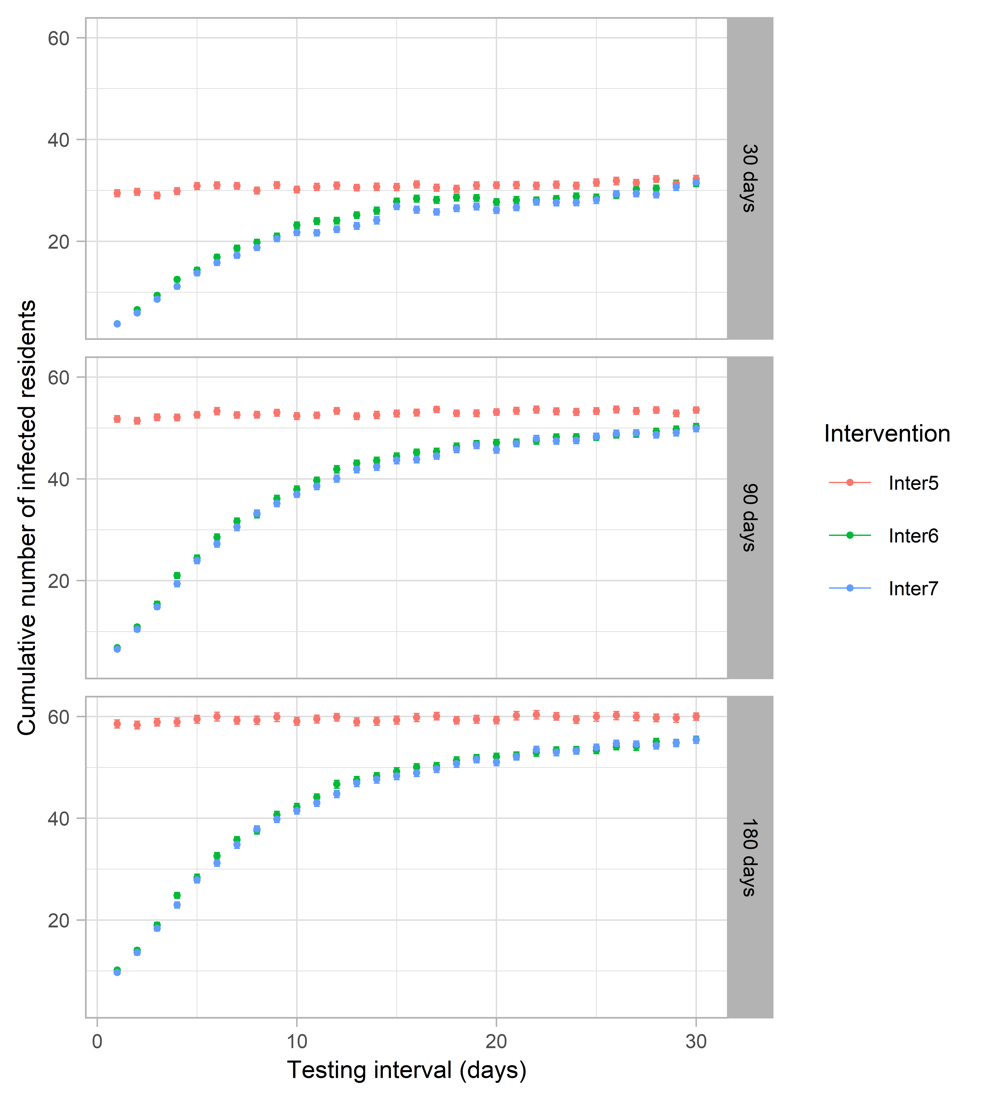


Figure S3-3: The impact of different testing intervals in routine testing scenarios on the cumulative number of infections after 30, 90, and 180 days

(Inter5: Inter1 + Weekly testing for residents; Inter6: Inter1 + weekly testing for staff; Inter7: Inter1 + weekly testing for staff and residents; Inter8: Inter6 + isolation upon admission; Inter9: Inter7 + isolation upon admission)

**APPENDIX S4: SENSITIVITY ANALYSES**

Table S4-1: Set up for uncertainty analyses of care home capacity, structure, and staff pool system

| Capacity (Total residents) | Number of units | Unit size (Residents per unit) | Care staff member per units per day | Staff pooling system |
| --- | --- | --- | --- | --- |
| *Base case model* | | | | |
| 80 | 2 | 40 | 16/15 | 33/32 staff members per unit |
| Capacity | | | | |
| 30 | 2 | 15 | 6 | 12 staff members per unit |
| 50 | 2 | 25 | 10 | 21 staff members per unit |
| 120 | 2 | 60 | 24 | 50 staff members per unit |
| *Number of units* | | | | |
| 80 | 1 | 80 | 31 | 65 staff members |
| 80 | 4 | 20 | 8 | 16 staff members per unit |
| 80 | 8 | 10 | 4 | 8 staff members per unit |
| *Residents per staff ratio*  *(Staff-resident contact rate is adjusted accordingly)* | | | | |
| 80 | 2 | 40 | 8 | 16 staff members per unit |
| 80 | 2 | 40 | 32 | 64 staff members per unit |
| *Staff pool system* | | | | |
| 80 | 2 | 40 | 16/15 | The pool of 65 staff members are shared between the two units |
| 80 | 2 | 40 | 16/15 | Unlimited |

Table S4-2: Outputs from Partial Rank Correlation Coefficient analyses

| Parameter | Inter1 | | Inter6 | |
| --- | --- | --- | --- | --- |
|  | **PRCC** | **p-value** | **PRCC** | **p-value** |
|  | **Outcome: Cumulative number of infected residents after 90 days** | | | |
| Test sensitivity | -0.05 | 3.50E-01 | -0.66 | 2.80E-39 |
| Resident leaving rate | -0.05 | 3.50E-01 | -0.09 | 1.15E-01 |
| Staff turnover | -0.05 | 3.50E-01 | -0.09 | 1.15E-01 |
| SD Compliance | -0.20 | 4.12E-04 | -0.35 | 6.18E-10 |
| Probability of symptomatic among infected staff | -0.55 | 5.78E-25 | -0.38 | 1.26E-11 |
| Probability of symptomatic among infected residents | -0.10 | 8.98E-02 | -0.08 | 1.93E-01 |
| Infection probability | 0.99 | 7.08E-267 | 0.99 | 2.47E-234 |
| Infection prevalence in the community | 0.70 | 3.81E-45 | 0.88 | 4.43E-77 |
| Infection prevalence in hospitals | 0.17 | 6.46E-02 | 0.00 | 9.38E-01 |
| Contact across units | -0.03 | 5.57E-01 | -0.01 | 8.39E-01 |
| Average staff-visitor contact rate | 0.24 | 2.61E-05 | 0.10 | 6.20E-02 |
| Average staff-staff contact rate | -0.12 | 3.06E-02 | -0.13 | 2.52E-02 |
| Average staff-resident contact rate | 0.85 | 5.25E-83 | 0.76 | 1.12E-58 |
| Average resident-resident contact rate | 0.22 | 1.57E-04 | 0.23 | 7.89E-05 |
| Average number of visitors per resident per day | 0.09 | 1.15E-01 | 0.09 | 1.03E-01 |
|  | **Outcome: Cumulative number of infected residents after 180 days** | | | |
| Test sensitivity | -0.08 | 1.44E-01 | -0.65 | 2.84E-37 |
| Resident leaving rate | -0.05 | 3.50E-01 | -0.08 | 1.15E-01 |
| Staff turnover | -0.05 | 4.11E-01 | -0.10 | 8.02E-02 |
| SD Compliance | -0.08 | 1.55E-01 | -0.32 | 7.12E-09 |
| Probability of symptomatic among infected staff | -0.36 | 1.55E-10 | -0.34 | 2.03E-09 |
| Probability of symptomatic among infected residents | -0.08 | 1.89E-01 | -0.10 | 7.66E-02 |
| Infection probability | 0.99 | 1.79E-241 | 0.99 | 1.54E-240 |
| Infection prevalence in the community | 0.77 | 8.58E-60 | 0.87 | 1.94E-94 |
| Infection prevalence in hospitals | 0.09 | 1.25E-01 | 0.03 | 5.90E-01 |
| Contact across units | -0.07 | 2.53E-01 | -0.01 | 8.24E-01 |
| Average staff-visitor contact rate | 0.38 | 6.35E-12 | 0.08 | 1.50E-01 |
| Average staff-staff contact rate | -0.21 | 3.44E-04 | -0.17 | 3.50E-03 |
| Average staff-resident contact rate | 0.77 | 4.54E-60 | 0.77 | 6.74E-61 |
| Average resident-resident contact rate | 0.20 | 4.74E-04 | 0.23 | 4.56E-05 |
| Average number of visitors per resident per day | 0.19 | 8.88E-04 | 0.21 | 2.61E-04 |

A negative value indicates a negative correlation – increasing the parameter decreases the outcome. A positive value indicates a positive correlation – increasing the parameter increases the outcome. In PRCC analysis in general, the parameters with large PRCC values (>0.5 or <– 0.5) and corresponding small p-values (<0.05) are deemed the most influential in the model.^51^


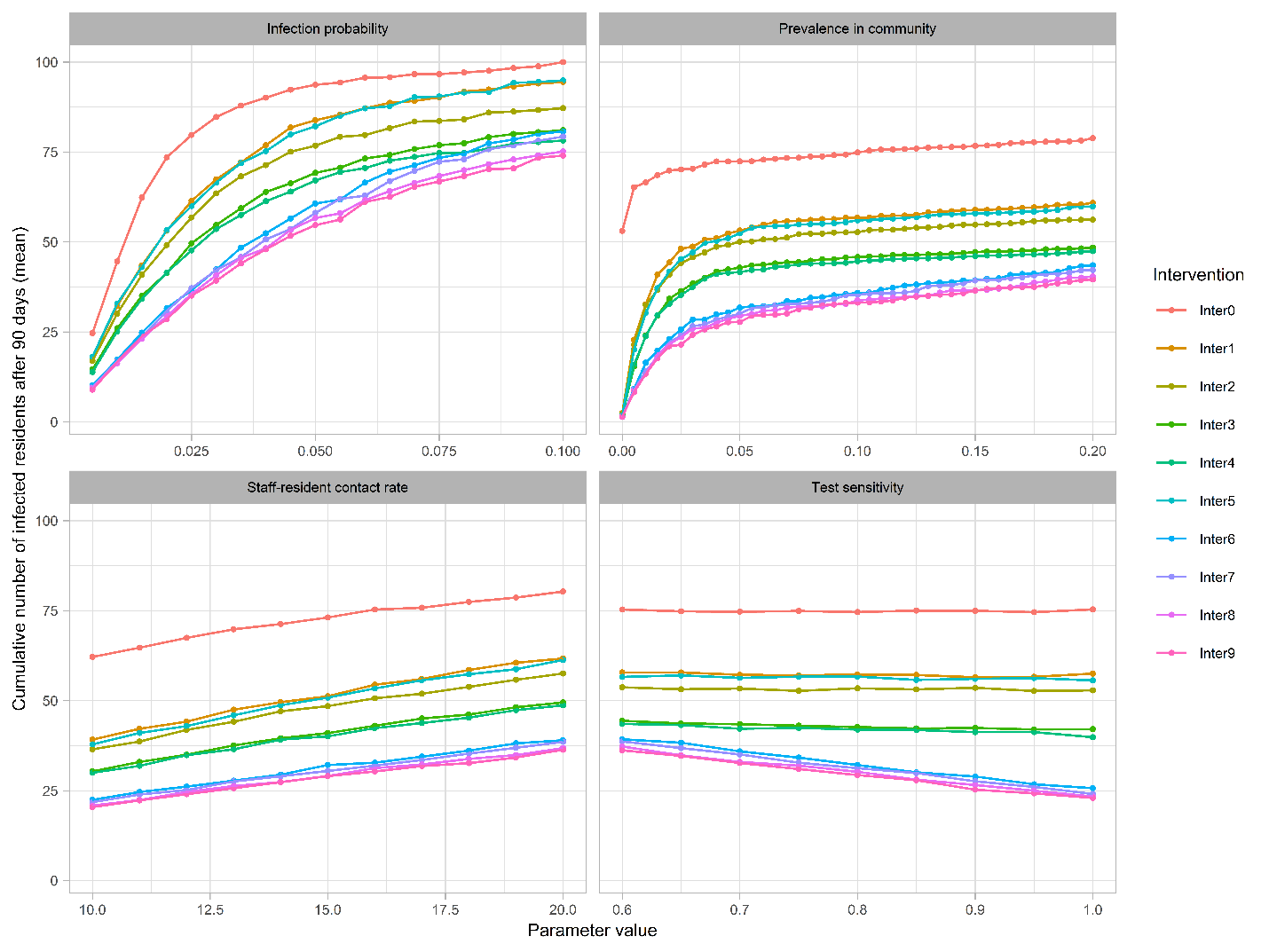


Figure S4-3: The impact of infection probability, infection prevalence in the community, staff-resident contact rate, and test sensitivity on the mean cumulative numbers of infected residents after 90 days in different intervention strategies over 300 simulations using the base case parameters
