# Supplementary material for: Evaluating intervention strategies in controlling coronavirus disease 2019 (COVID-19) spread in care homes: An agent-based model
Source: Infect Control Hosp Epidemiol. 2020 Dec 14:1–11. doi: 10.1017/ice.2020.1369 (PMC7783094; doi:10.1017/ice.2020.1369)
Supplement: Supplementary file 1 [file S0899823X20013690sup.zip › S0899823x20013690sup002.zip!/ICE-2001369_Supplementary_files/41023_Nguyen_OA_COV_FIGURES.docx]

**Figure 1**

**Figure 2**

***Figure 3***

**Figure 4**

**Figure 5**
